# Imagery Rescripting on Guilt-Inducing Memories in OCD: A Single Case Series Study

**DOI:** 10.3389/fpsyt.2020.543806

**Published:** 2020-09-30

**Authors:** Katia Tenore, Barbara Basile, Teresa Cosentino, Brunetto De Sanctis, Stefania Fadda, Giuseppe Femia, Andrea Gragnani, Olga I. Luppino, Valerio Pellegrini, Claudia Perdighe, Giuseppe Romano, Angelo M. Saliani, Francesco Mancini

**Affiliations:** ^1^Associazione Scuola di Psicoterapia Cognitiva (APC-SPC), Rome, Italy; ^2^Department of Social and Developmental Psychology Sapienza, University of Rome, Rome, Italy; ^3^Department of Human Sciences, Marconi University, Rome, Italy

**Keywords:** obsessive-compulsive disorder, imagery rescripting, memories, criticism, guilt

## Abstract

**Background and objectives:**

Criticism is thought to play an important role in obsessive-compulsive disorder (OCD), and obsessive behaviors have been considered as childhood strategies to avoid criticism. Often, patients with OCD report memories characterized by guilt-inducing reproaches. Starting from these assumptions, the aim of this study is to test whether intervening in memories of guilt-inducing reproaches can reduce current OCD symptoms. The emotional valence of painful memories may be modified through imagery rescripting (ImRs), an experiential technique that has shown promising results.

**Methods:**

After monitoring a baseline of symptoms, 18 OCD patients underwent three sessions of ImRs, followed by monitoring for up to 3 months. Indexes of OCD, depression, anxiety, disgust, and fear of guilt were collected.

**Results:**

Patients reported a significant decrease in OCD symptoms. The mean value on the Yale−Brown Obsessive Compulsive Scale (Y-BOCS) changed from 25.94 to 14.11. At the 3-month follow-up, 14 of the 18 participants (77.7%) achieved an improvement of ≥35% on the Y-BOCS. Thirteen patients reported a reliable improvement, with ten reporting a clinically significant change (reliable change index = 9.94). Four reached the asymptomatic criterion. Clinically significant changes were not detected for depression and anxiety.

**Conclusions:**

Our findings suggest that after ImRs intervention focusing on patients’ early experiences of guilt-inducing reproaches there were clinically significant changes in OCD symptomatology. The data support the role of ImRs in reducing OCD symptoms and the previous cognitive models of OCD, highlighting the role of guilt-related early life experiences in vulnerability to OCD.

## Introduction

Obsessive-compulsive disorder (OCD) is a common clinical condition experienced by about 1.2% of the population and with an estimated lifetime prevalence of 2.3% (1, 2). OCD produces suffering and seriously compromises patients’ overall quality of life, weighing heavily also on the quality of life of the co-habiting family (3–6).

OCD is characterized by obsessions and compulsions. Obsessions are “*recurrent and persistent thoughts, urges, or impulses that are experienced at some time during the disturbance, as intrusive and unwanted, and that in most individuals causes marked anxiety or distress”*. Compulsions are *“repetitive behaviors … or mental acts … that the individual feels driven to perform in response to an obsession or according to rules that must be applied rigidly. The behaviors or mental acts are aimed at preventing or reducing anxiety or distress, or preventing some dreaded event or situation”* (7).

A crucial role in OCD onset and maintenance has been attributed to responsibility and guilt by Rachman (8–10) and by Salkovskis (11). Results from different studies have corroborated this thesis. OCD patients experience more intense guilt and higher responsibility when compared to other people (12–17). OCD patients are characterized by high levels of fear of guilt (18–20). Takahashi et al. (21) found similar brain activity between OCD patients when exposed to stimuli eliciting OCD symptoms, and nonclinical subjects when exposed to stimuli eliciting guilt. Moreover, studies have corroborated the hypothesis that compulsions are aimed at reducing or preventing responsibility and guilt. Lopatcka and Rachman (22) and Shafran (23) have shown that OCD symptoms diminish when the level of responsibility is lowered, by asking to put an agreement in writing, so the responsibility for any consequence for not carrying out the compulsions was of the experimenter or by varying the presence or absence of the experimenter during the behavioral task. Cognitive Therapy Interventions (e.g., Socratic dialogue, pie-technique, double-standard technique, and the court technique) aimed at reducing the responsibility and consequentially the risk of being guilty (24–26) lead to a significant reduction of OCD symptoms. Additionally, when responsibility and fear of guilt are induced experimentally, especially when associated with the fear of making mistakes, nonclinical participants begin to behave in an obsessive-compulsive–like way and those with OCD show an increase in obsessive-compulsive behaviors (16, 18, 27–29). Arntz and colleagues (30) experimentally induced the sense of responsibility and the fear of guilt in OCD patients, in other-clinical and nonclinical groups. Checking behaviors were higher in OCD patients than in the other two groups. This result suggest that OCD patients, regardless the subtype, are particularly sensitive to responsibility and fear of guilt. One might ask if checking behaviors are aimed at reducing or preventing responsibility and guilt, while washing behaviors are only aimed at reducing or preventing disgust and not responsibility and guilt. According to Bhikram et al. [(31), 300] “*exaggerated and inappropriate disgust reactions may drive some of the symptoms of OCD, and in some cases, may even eclipse feelings of anxiety*.” Two questions arise: What is the relationship between guilt and disgust? Is it possible that guilt implies the activation of disgust resulting in washing behavior? Some studies (32, 33) found the so-called Macbeth effect “*that is, a threat to one’s moral purity induces the need to cleanse oneself … physical cleansing alleviates the upsetting consequences of unethical behavior and reduces threats to one’s moral self-image*.” [(32), 1451]. This effect has not been detected in some studies (34), but Reuven et al. (35) found it particularly prominent in OCD. Ottaviani et al. (36) found that in nonclinical participants, the induction of a specific sense of guilt, the deontological guilt, which is related to having transgressed moral norms, regardless of whether someone has been harmed (37, 38) elicits obsessive-like washing behaviors, which reduce guilt and increase positive emotions (39).

It is plausible, therefore, that all obsessive symptomatology, not only checking compulsions, are the expression of an intense concern for one’s own morality, in particular for the deontological morality (37, 38, 40).

Such moral concern is found in Ehntholt’s and colleagues work (41, 779):

*“OCD patients reported more fear that others would see them in a completely negative manner, e.g., others would “loathe” or “despise” them if it was possible that they would cause others harm or problems, suggesting a sensitivity to blame and criticism. Our findings that those in the OCD group are more sensitive to the criticism of others is also consistent with Turner, Steketee & Foa (*1979*)”*.

In line with these results are those from a small pilot study from Mancini and colleagues (42), where OCD participants, compared to non-OCD, showed higher distress when exposed to Ekman’s Pictures of Facial Affect of contempt, anger, disgust, if requested to imagine that such expressions were addressed to them and, above all, that they deserved them. Moreover, OCD participants declared, more than other participants, that they reminded them the faces of the parents, or one of the two, and their parents’ facial expressions at a time when they were being reproached and experiencing intense distress. In fact, families of obsessive patients are described as demanding and critical [see (43–45)]. In a recent study, Basile et al. (46) found that OCD patients reported significantly more painful memories of guilt-inducing blame/reproach compared to a non-OCD group.

An interesting observation of the type of discipline used by parents of future OCD patients is the threat to the continuity of the relationship itself (47). Clinical observations show that in cases of reproach, parents of future OCD patients withdraw love, ignore the child and are not prone to forgive (45). It is plausible that these experiences have taught the patient that a small mistake is enough to receive serious, aggressive, contemptuous, demeaning reproaches by significant figures such as parents, without having the possibility to justify oneself or be forgiven, and that his/her behavior can determine the end of such significant relationships (45). Briefly, the expectation that guilt has catastrophic consequences may derive from these kinds of experiences. Along the same lines, according to Pace et al. (43), obsessive behaviors may be considered as strategies used by the child to avoid criticism and obtain approval. According to Cameron (48), obsessive behaviors may be created as methods to obtain the parents’ satisfaction and avoid being criticized. Some studies suggest that obsessions could also be intrusive mental images that evoke adverse early experiences (49), and that obsessive thoughts have implications for a person’s sense of self (50) as well as such guilt-inducing experiences.

It is possible to modify the meaning attached to past adverse or traumatic events, especially childhood or adolescence events, intervening in those events’ memories through imagery rescripting (ImRs). ImRs is an experiential technique that has shown promising results in different clinical disorders (51, 52). It has been theorized that the way ImRs works is by changing the meaning attached to memories (53).

ImRs has been employed in OCD by Veale et al. [(54), 230] who stated that:

“Cognitive Behavioral Therapy (CBT), including exposure and response prevention, remains the psychological treatment of choice for Obsessive-Compulsive Disorder … However, a significant proportion of cases still fail to respond to CBT … This has prompted the search for new target areas for intervention, in the hope that outcomes can be improved.”

Veale et al. (54) examined the efficacy of one single ImRs session, as a standalone intervention, where intrusive images linked to aversive memories were present. The presence of intrusions linked to aversive past events has been detected in many studies (49, 55, 56). In the study of Veale et al. (54) after ImRs, nine patients showed a reliable change and seven a clinically significant change at the 3-month follow-up session. A major change was detected three months after the end of treatment. More recently, Maloney et al. (57) investigated the efficacy of ImRs as a treatment for OCD cases that were not responsive to standard exposure and response prevention. In the study, the authors investigated the efficacy of 1–6 ImRs sessions in 13 OCD patients who experienced intrusive distressing images associated with OCD. Of those 13 patients, 12 reached an improvement of at least 35% in OCD symptoms. Six patients reached the improvement after only a single ImRs session, whereas the rest required 2–5 ImRs sessions. The results of both studies were very promising, suggesting the opportunity to carry out other studies on ImRs’ efficacy on OCD.

Starting from the work of Veale et al. (54) and considering the role of guilt-inducing reproaches in the development of the fear of guilt, we hypothesize that an intervention of ImRs on childhood memories of guilt-inducing reproach in OCD people could reduce current obsessive symptoms.

The main hypothesis that we wanted to test is that after an intervention of ImRs, OCD symptoms—regardless the subtype—would decrease and that change would be maintained.

We also hypothesize a reduction in both the fear of guilt and in the propensity to disgust.

In addition we measured the effect of ImRs on anxiety and depression, to control the effect of ImRs on these two emotions. We expected that the effect of ImRs would be less than the one on specific obsessive symptoms, due to the specific nature of ImRs intervention on memories for OCD.

## Method

The study is centered on a single-case series experimental design. According to Lobo et al. (58), in single-case studies, indexes are assessed repeatedly for each participant across time. The different interventions are defined as “phases,” and one phase is considered as a baseline for comparison. In single case studies, a control group is not required because each participant represents a proper control.

### Participants

A sample of 18 participants seeking treatment for OCD at “Studio di Psicoterapia Cognitiva” in Rome was enrolled for the study. At an early stage, recruitment was attempted through the Internet and flyers’ announcements, but these modalities were ineffective.

Twenty-four people, seeking treatment voluntarily, were asked to enroll in the study and two refused to take part. Of the 22 participants who accepted, 18 completed the procedure, two dropped out and two were excluded due to a change of psychopharmacological drugs during the procedure.

Approximately two thirds had received prior treatment and were not awaiting other treatments, but a few started a treatment after the last follow-up. Nobody was in treatment during the 9 months of the experimental trial.

The participants were not preselected for showing a relevant memory, but all showed at least one memory, **Table 1** reports gender, age, disorder duration in years, and OCD subtypes for each subject. Mental contamination refers to that form of contamination arising from “*experiencing psychological or physical violation. The source of the contaminations is a person, not contact with an inert inanimate substance*” (4, 59).

**Table 1:** Clinical summary of participants.

Participant	Gender	Age	Disorder Duration in Years	OCDSubtype
1	M	25	10	Ruminations/Intrusive Thoughts
2	M	39	4	Contamination/Mental Contamination
3	M	28	12	Contamination/Mental Contamination
4	M	36	16	Ruminations/Intrusive Thoughts
5	M	28	13	Contamination/Mental Contamination
6	M	27	13	Checking
7	F	52	25	Ruminations/Intrusive Thoughts
8	F	31	9	Contamination/Mental Contamination
9	F	42	9	Contamination/Mental Contamination
10	M	38	2	Ruminations/Intrusive Thoughts
11	F	28	20	Contamination/Mental Contamination
12	F	46	36	Contamination/Mental Contamination
13	M	32	12	Checking
14	F	24	13	Ruminations/Intrusive Thoughts
15	F	46	18	Checking
16	F	37	15	Checking
17	F	34	20	Ruminations/Intrusive Thoughts
18	F	24	3	Ruminations/Intrusive Thoughts

### Inclusion Criteria

Participants were included if they were aged 18–65 years with an OCD diagnosis according to DSM-5 (7) and a score on the Yale–Brown Obsessive Compulsive Scale (Y-BOCS) higher than 18.

### Exclusion Criteria

Patients were excluded if they were having ongoing psychotherapy, if psychotherapy had ended less than three months prior to the beginning of the procedure or if psychopharmacological drugs had been changed in the last three months or during the procedure.

To monitor for possible changes in drug therapy, at each assessment meeting the participants were asked whether the therapy had remained constant. The participants received the same procedure but if there was a change in drugs their data were not considered in the analysis because we could not be sure whether the effect on symptoms was attributable to the intervention or to the change in drugs.

A further exclusion criterion was a comorbid diagnosis of psychosis, schizotypy, mania, borderline personality disorder, alcoholism, impaired cognitive function (assessed on the basis of the educational level and with a clinical interview) or dissociation symptoms [a score higher than 30 on the Dissociative Experiences Scale: (60)].

### Measures

1. *The Structured Clinical Interview for DSM-5* [SCID-5; (61)] is a clinician-administered semi-structured interview, aimed at assessing diagnoses according to the fifth edition of the DSM [DSM-5; (7)].
2. The *Dissociative Experiences Scale* [DES; (60)].
  The DES is a 28-item self-report questionnaire that assesses forms of dissociation. The scores range from 0 (never) to 100 (always). The DES has proven to have adequate test–retest reliability as well as a good internal consistency, and good clinical validity (62, 63). A cutoff score of 30 to detect dissociative psychopathology in clinical sample is recommended (64, 65).
3. *The Yale–Brown Obsessive-Compulsive Scale* [Y-BOCS; (66)].
4. The Y-BOCS is a 10-item clinician-rated scale that assesses the severity of obsessive-compulsive symptoms and the effectiveness of treatment. The clinician attributes a score from 0 (absence of symptoms) to 4 (very severe symptoms). The total score is in a range from 0 to 40. Higher scores indicate more severe OCD symptomatology. The scale has proven to have high internal consistency [alpha = 0.82; (67)].
5. *The Obsessive-Compulsive Inventory* [OCI-r; (68)].
  The OCI-r is an 18-item self-report questionnaire, which assesses the severity of OC symptoms on the 5-point Likert scale. There are six subscales (washing, checking, ordering, obsessing, hoarding, and mental neutralizing). The total score ranges from 0 to 72. The OCI-r Italian version (67) showed good internal consistency as well as a convergent and divergent, and criterion validity [alpha = 0.85; (67)].
6. The *Beck Depression Inventory-Second Edition* [BDI-II; (69)]. The BDI-II is a 21-item self-report, measuring the severity of several components of depression. The Italian version of the BDI-II has proven to have good internal consistency [alpha = 0.80; (70)] as well as good convergent and divergent and criterion validity (70, 71).
7. The *Beck Anxiety Inventory* [BAI; (72)]. The BAI is a 21-item self-report, that measures the severity of anxiety. The BAI Italian version shows good internal consistency [alpha = 0.89; (70)] as well as good convergent and divergent, and criterion validity (70, 73).
8. The Fear of Guilt Sclale [FOGS; (19, 20)]. FOGS is a 17-item self-report scale, ranging from 0 to 7, assessing the extent to which a person values and fears guilt and how she/he behaves in relation to guilt. The FOGS consists of two factors: Punishment (drive to punish oneself for feelings of guilt) and Harm Prevention (drive to proactively prevent guilt). The FOGS demonstrated strong internal consistency as well as convergent and divergent validity [alpha = 0.92; (20)]. It also significantly predicted OCD symptom severity over measures of neuroticism, depression, trait guilt, and inflated responsibility beliefs (19).
9. The *Disgust Propensity Questionnaire* [DPQ; (74)]. DPQ is a 33-item scale aimed at assessing the individual’s propensity for disgust. The participant expresses the agreement on a 5-point Likert scale from 0 (“not at all”) to 4 (“very much”). The total score range is from 0 to 132. The questionnaire has been proven to have a one-factor structure, as well as good internal consistency [alpha in the range 0.85–0.91; (74)] as well good test–retest reliability (ICC = 0.85) and also construct validity (74).

### Procedure

Participants who accepted to be enrolled in the study signed an informed consent form. In an initial clinical interview, we checked for inclusion and exclusion criteria. The inclusion criteria were assessed through clinical interview and the Structured Clinical Interview for DSM-5 (SCID-5: 61). Diagnostic interviews were conducted by experts who had a master’s degree in psychodiagnosis, were trained to administer the SCID and conducted the interview according to the reference manuals; they were also blind to the study’s hypothesis. In the second session we measured the obsessive symptoms’ subtype and severity; and in the third meeting we ran an *ad hoc* interview on memories (see Appendix) in order to detect guilt-inducing reproaches memory that could be examined in the three following ImRs sessions. The selection of the memories was driven by the aim of intervening on generic memories of guilt-inducing reproaches not necessarily related to the current symptomatology. We selected memories in a different way from Veale et al. (54), where the authors selected participants who experienced intrusive imagery as part of their OCD, which was considered by the participant and assessor to be emotionally linked to memories of past aversive events, and from Maloney et al. (57), where intrusive imagery was selected because it was associated with OCD and considered by the patient to be linked to memories of aversive events.

We asked participants to recall reproaches similar to those which had been found by Basile et al. (46).

As already stated, we found, for each participant, generic memories of guilt-inducing reproaches, and so no one was excluded for this reason.

In particular, we focused on generic reproach experiences not necessarily related to the symptom domain. For example, a childhood memory selected by an OCD patient with washing symptoms was not directly related to being reproached for being dirty, but rather was independent of the symptom domain. The first criterion used for the memory’s selection was the earliest childhood memories reported by the participants, the second was the most intense memory from an emotional point of view.

Participants received a symptoms’ assessment and then as Veale and colleagues (54) did were randomized to 4, 8, 12, or 16 days of symptom monitoring before receiving ImRs (4 participants in the condition of 4 days monitoring, 5 in the condition of 8 days monitoring; 4 in the condition of 12 days monitoring; 5 in the condition of 16 days monitoring). Within the three 45-min ImRs treatment sessions, the previously selected memory was addressed and rescripted. For each participant, we selected one memory that was rescripted during the three sessions. The clinicians who ran the ImRs sessions were all experts in cognitive-behavioral therapy (CBT) for OCD (with an average of 10 years of experience) and in imagery techniques and the adherence to the protocol was supervised by three trainers and supervisors in ImRs. Based on the work of Veale et al. (54), we carried out each ImRs session according to Arntz’s three-stage technique (75), adapting it to the Schema Therapy suggestions (76) for patients with difficulty meeting their needs autonomously. The technique consisted of a first phase in which the patient was invited to relive the memory with his/her eyes closed, from the standpoint of their childhood self. In the second phase, the patient looked at the same event as an adult, tried to detect the unmet need of his/her childhood self and proposed an imaginative change (the rescripting) aimed at satisfying the unmet emotional needs. In the third phase, the patient as a child looked at the event with the changes proposed by the adult. In line with the procedure, if the patient could not find a solution to the unmet need in the second phase, the therapist then suggested some interventions or asked the patient to include the therapist into the image of their childhood, in order to meet the patient’s needs. The traditional protocol was employed as proposed by Arntz—“*part of rescripting involves a secure adult that meets the child’s needs to be reassured and comforted*” [(53), 467]. By unmet need we mean the core emotional need, whose unfulfillment is the cause of the emotional sufferance. The intervention of the adult in the second phase, and the rescripting, are stimulated by the clinician’s questions: “Is there anything you would like to do?” “Is there anything that should be done?”

After each session, as per the traditional protocol (53, 77), the patient listened to a recording of the session between one session and the next. Data were not collected between sessions. After clinical intervention, four follow-up assessment sessions (at 7, 30, 60, and 90-day intervals, respectively) were held, as in Veale et al. (54).

**Figure 1:**
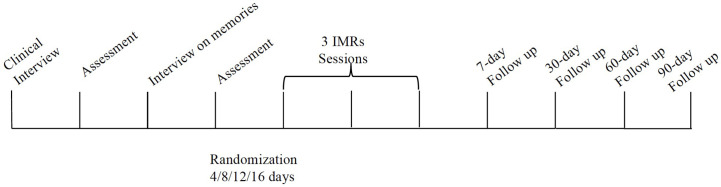
Procedural timeline.

An outline of the procedure is shown in **Figure 1** and the procedure has been approved by the ethical committee of Guglielmo Marconi University.

**Appendix Table 1** shows a summary of the contents of the rescripted memories. It reports the event and the emotion experienced, the participant’s age in the memory and meaning attached to the memory, verbally expressed by the participant. The “Emotion and meaning of memory” refers to the answers that participants gave when, in the first phase of rescripting, the patient was invited to relive the memory with his/her eyes closed, from the standpoint of their childhood self. In that phase of the technique they were asked: “What is happening?” “What do you feel?” “What do you think about the situation?” and the therapist just wrote down the participant’s verbal expression. “New meaning” after the ImRs refers to the event’s appraisals after the third intervention and the answers to the question the therapist asks “What do you think about the situation?,” which is asked in the third phase of rescripting, when the patient as a child looked at the event with the changes proposed by the adult in the second phase of the technique.

### Statistical Analysis

Data were analyzed using the Statistical Package for Social Science (SPSS 25, Inc., Chicago, IL) for parametric and nonparametric analyses, while the Leeds Reliable Change Indicator was used to calculate change indexes (78, 79).

Beyond initial descriptive analyses, we calculated reliable and clinically significant change indexes for all clinical measures (Y-BOCS, OCI-r, BAI, and BDI-II). Like Veale and colleagues (54), we considered the over-time change of the Y-BOCS total score. In particular, we considered indexes related to (a) reliable change and (b) clinically significant change (80). We evaluated the change in scores from screening to 90-day follow-up of at least 2 standard deviations (SDs) from the original mean. A reliable change was identified by the Leeds Reliable Change Indicator as a 10-point reduction on the Y-BOCS. A clinically significant change is the condition where criterion a was satisfied and the participant’s scores were under the clinical cutoff (for the Y-BOCS, score less than 17). As proposed by Veale and colleagues (54), we considered Pallanti’s asymptomatic criterion (81), which refers to an approximate total absence of OCD symptoms. The asymptomatic criterion for OCD has been defined as a recovery on the Y-BOCS (score 7 or less). The same analysis was performed for the OCI-r total score.

Paired samples t-tests and Wilcoxon signed-rank tests on the different measures (e.g., Y-BOCS, OCI-r, BAI, and BDI-II) were also performed between screening and 90-day follow-up, as well as, between pre-ImRs baseline and 90-day follow-up.

Afterward, two distinct linear mixed regression models were performed in order to test the fixed effect of the ImRs treatment on the OCD-related measures (i.e., Y-BOCS and OCI-r) and its random variations across patients. The strength of these kinds of models’ lies in the fact that the random variability of the parameters is also taken into account. Thus, the analysis allowed us to estimate whether the OCD-related symptoms decreased after the ImRs intervention and across the different measurement times, simultaneously considering the random variability of such hypothesized reduction for each of the 18 patients. Before running the analyses, it was necessary to carry out a restructuring of the data. Thus, we changed the data matrix from a wide format to a long format. Afterward, we stacked the scores of the Y-BOCS and OCI-r, obtained at each measurement time, into two distinct variables. These variables were in turn associated with an indicator of the measurement times (i.e., pre-ImRs baseline, 7-, 30-, 60-, and 90-day follow-up). Since we were interested in testing the effectiveness of the ImRs intervention, we focused our attention on the observed changes starting from the pre-ImRs baseline. Thus, the indicator variable was centered on the pre-ImRs assessment by coding such time point as 0. In this way, we were able to test the fixed effect of the time and the related random variability of intercept and slope. Moreover, we also estimated the quadratic effect of the time to test whether the differences that had emerged were in the extremes of the experimental region or inside it. These analyses were performed with the lme4 package (82) using RStudio (83), a graphical interface for R software. Both models were tested using a restricted maximum likelihood method (REML).

Then, a mixed ANOVA was conducted to determine the extent to which levels of change on fear of guilt (low vs. high) affected ImRS intervention on obsessive symptoms.

Finally, we computed intercorrelations among all the variables investigated at the 90-day follow-up in order to explore the relationships among them after the ImRs intervention.

## Results

First, we explored the structure of our data by means of some descriptive statistics (see **Table 2**). Thus, we computed the mean and the related standard deviations of each measure at the different detection times. Moreover, because of the reduced size of the sample under examination, we also computed the median and considered the interquartile range as a measure of the data dispersion from their central value.

**Table 2:** Descriptive statistics: Means, Standard Deviations, Median and Inter-Quartile Range (IQR) of the Yale–Brown Obsessive–Compulsive Scale (Y-BOCS), Obsessive–Compulsive Inventory revised (OCI-r), Beck Depression Inventory - Second Edition (BDI-II), Beck Anxiety Inventory (BAI), Fear of Guilt Scale (FOGS), Disgust Propensity Questionnaire (DPQ), Dissociative Experience Scale (DES).

Measures	Statistics	Screening	Pre-ImRs	7-day follow-up	30-day follow-up	60-day follow-up	90-day follow-up (Post treatment)
**Y-BOCS**	*Mean*	25.9	24.2	18.4	17.8	15.1	14.1
	*Sd*	5.7	8.8	6.8	5.9	7.1	5.9
	*Median*	26	23	22	19	17.5	12.5
	*IQR*	10	9	10	5	9	7
**OCI-r**	*Mean*	28.3	28.4	19.9	20.1	19.6	19.7
	*Sd*	12.6	14.3	15.7	17.8	17.1	17.6
	*Median*	30	30	19.5	18	16.5	15.5
	*IQR*	17	13	15	17	16	14
**BDI-II**	*Mean*	21.4	20.3	16.4	14.3	14.6	14.4
	*Sd*	11.4	10.2	11.9	12.2	12.8	13.5
	*Median*	17	17	14	10.5	11.5	10.5
	*IQR*	23	19	17	14	16	18
**BAI**	*Mean*	23.5	21.1	16.7	15.1	14.5	13.1
	*Sd*	14.9	13.0	12.4	11.7	12.7	9.9
	*Median*	18.50	19	13	11.5	10.5	8
	*IQR*	29	23	17	17	18	13
**FOGS**	*Mean*	75.8	–	–	62.9	–	65.0
	*Sd*	18.2	–	–	20	–	20.3
	*Median*	223.50	–	–	164	–	200
	*IQR*	89	–	–	68	–	77
**DPQ**	*Mean*	17.9	–	–	–	–	16.2
	*Sd*	8.9	–	–	–	–	8.7
	*Median*	21.50	–	–	–	–	16.5
	*IQR*	15	–	–	–	–	13
**DES**	*Mean*	13.4	–	–	–	–	–
	*Sd*	14.8	–	–	–	–	–
	*Median*	9.6	–	–	–	–	–
	*IQR*	15.8	–	–	–	–	–

### Clinical Response to Imagery Rescripting

At the 3-month follow-up, 14 of the 18 participants (77.7%) achieved an improvement of ≥35% on the Y-BOCS, defined by Farris (84) and Mataix-Cols et al. (85) as corresponding to the most predictive of treatment response. Based on the results of the retrospective investigation of Tolin et al. (86), that is, the reduction criterion of at least 30% on the Y-BOCS as optimal for determining clinical improvement, it is possible to say that 15 participants (83%) reported a significant improvement.

Eleven of the 18 participants (61%) reached an absolute raw score of 12 or less on the Y-BOCS measure, which is identified by Lewin et al. (87) as optimal for predicting remission in a clinical setting. Based on Pallanti’s asymptomatic criterion (81), four participants reached the asymptomatic criterion (7 or less on Y-BOCS) at 90-day follow-up.

#### Reliable and Clinically Significant Change on the Y-BOCS

Of the whole sample, 13 patients reported a reliable change, with 10 of them revealing a clinically significant change on the OCD clinical measure (RCI = 9.94) using criterion A. The average scores from pretreatment and post-treatment met the criteria for reliable and clinically significant change.

**Figure 2:**
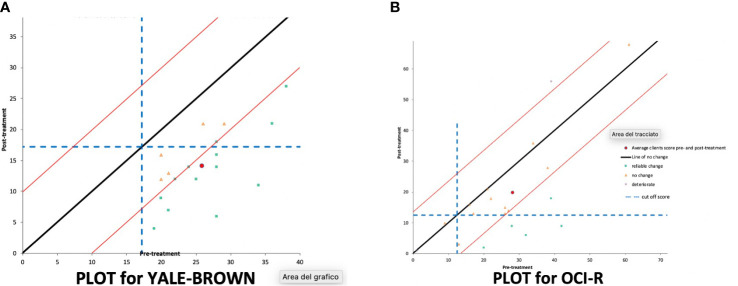
Reliable and clinically significant change for **(A)** Y-BOCS (Yale–Brown Obsessive Compulsive Scale) and **(B)** for OCI-r (Obsessive-Compulsive Inventory revised) at 90-day follow-up.

**Figure 2A** reports single participants’ pretreatment Y-BOCS scores on the x-axis and post-treatment scores on the y-axis. Participants, who were in the lower-right quadrant and under the parallel lines achieved a reliable and clinically significant change.

#### Reliable and Clinically Significant Change on the OCI-r

At the 90-day follow-up, considering the OCI-r, 12 patients showed no significant improvement, 1 deteriorated (reached 17 points on the scale), while five participants reliably improved, with four of them showing a clinically significant change (RCI = 13.49), using criterion C. According to Jacobson and Truax (80), if you do not have an externally determined cut score you can use one based on statistical criteria. Criterion C is the one suggested, when clinical and comparison groups’ norms overlap. The average scores from pretreatment and post-treatment did not meet the criteria for reliable and clinically significant change.

**Figure 2B** reports single participants’ pretreatment OCI-r scores on the x-axis and post-treatment scores on the y-axis. Participants, who were in the lower-right quadrant and under the parallel lines achieved a reliable and clinically significant change.

#### Reliable and Clinically Significant Change on the BDI-II and BAI

Of the total sample, nine patients did not show any improvement in depressive symptoms as assessed with the BDI-II (RCI = 11.36). One deteriorated, eight reliably improved, and six showed clinically significant change. When assessing for any clinically significant change on the BAI, 12 showed no improvement, and 6 improved (RCI = 11.69). The average scores from pretreatment and post-treatment did not meet the criteria for reliable and clinically significant change.

#### Parametric and Nonparametric Comparisons

In order to obtain an estimate of the reduction of the scores on the examined measures after the ImRs treatment, we implemented both parametric and nonparametric tests. As parametric test, we conducted several paired samples t-tests. The comparisons concerned the scores obtained by the patients at the screening phase and at the 90-day follow-up on all the measures used in the study, except for the Dissociative Experiences Scale (60). **Table 3** clearly shows that at the 90-day follow-up (vs. Screening) there are significant reductions in all the measures considered, and that these significant decreases are accompanied by remarkable effect sizes. A unique exception was represented by the comparison concerning the DPQ, which turned out to be only marginally significant.

**Table 3:** Paired samples t-test for the scores at the Screening detection time compared to 90-day follow-up and for the scores at pre-ImRs baseline compared to 90-day follow-up.

Measures	Comparison	*Mean Diff*.	*Sd*	*se*	*t*	*df*	*p*	*95%CI*		*Effect size (d)*
								*Lower*	*Upper*	
**Y-BOCS**	Screening vs. 90 days	11.8	4.9	1.7	10.1	17	.001	9.35	14.31	2.05
	Pre-ImRs vs. 90 days	9.5	6.5	1.6	6.04	16	.001	6.15	12.79	1.08
**OCI-r**	Screening vs. 90 days	8.6	12.3	2.9	2.96	17	.009	2.47	14.75	0.63
	Pre-ImRs vs. 90 days	7.6	12.3	2.9	2.54	16	022	1.26	13.91.	0.53
**BDI-II**	Screening vs. 90 days	7.1	10.8	2.5	2.77	17	.013	1.69	12.42	0.61
	Pre-ImRs vs. 90 days	5.4	8.9	2.2	2.47	16	.025	0.76	9.95	0.52
**BAI**	Screening vs. 90 days	10.4	12.1	2.8	3.67	17	.002	4.45	16.44	0.70
	Pre-ImRs vs. 90 days	8.2	6.9	1.7	4.85	16	.001	4.64	11.83	0.63
**FOGS**	Screening vs. 90 days	36.7	70.9	16.7	2.19	17	.042	1.40	71.94	0.59
**DPQ**	Screening vs. 90 days	3.2	6.01	1.6	2.061	14	.058	−0.13	6.53	0.19

Furthermore, we also tested further comparisons between the scores obtained on the clinical measures of Y-BOCS, OCI-r, BDI-II, and BAI at the pre-ImRs baseline and at the 90-day follow-up. This analysis was therefore more focused on the effectiveness of the ImRs treatment. As expected, our hypotheses were corroborated: The paired samples t-tests revealed a significant decrease in scores on the four clinical measures. Also, in this case the reductions were associated with an effect of noticeable magnitude. The effect size of each paired samples t-test was computed by dividing the emerged differences by the standard deviation of the interested baseline. As highlighted by Morris (88), this procedure provides more reliable effect size estimates compared to using post-test or pooled standard deviation as denominator.

The results that emerged from the t-tests therefore seemed to provide empirical evidence about the effectiveness of the ImRs treatment. Given the relatively small number of participants, in order to provide some evidence to the robustness of the findings, we ran a post-hoc power analysis on the t-test conducted in the study, by using GPower. Specifically, we implemented a post-hoc power analysis for testing difference between two dependent means (matched pairs). By setting a medium effect size (Cohen’s d) of 0.7, error probability of 0.05, and two tailed distribution, the analysis revealed a statistical power of 0.80 associated to the sample size of 18 participants.

Moreover, we tried to provide further support and robustness to our results through a nonparametric test. Thus, we implemented a Wilcoxon signed-rank test for the nonparametric comparison of the Y-BOCS, OCI-r, BDI-II, and BAI scores between the pre-ImRs baseline and at the 90-day follow-up. As can be seen in **Table 4**, results were consistent with those of paired samples t-tests. Specifically, the Wilcoxon tests showed a decrease of both scores of Y-BOCS and BAI for 16 participants, as well as, a reduction in the scores of BDI-II and OCI-r for 12 and 11 patients, respectively. Moreover, all test statistics were associated with an effect size (r) between medium and high values. These effect sizes were computed by dividing the z test statistic by the square root of the total number of observations (89).

**Table 4:** Wilcoxon signed-rank test (nonparametric).

Measures	Negative Ranks	Positive Ranks	Ties	Total	Z	p	Observations	Effect Size (r)
**Y-BOCS**	16	0	1	17	−3.52	.001	34	−.60
**OCI-r**	11	4	2	17	−2.10	.035	34	−.36
**BDI-II**	12	3	2	17	−2.39	.017	34	−.41
**BAI**	16	0	1	17	−3.52	.001	34	−.60

#### OCD Scores in the Different Protocol Phases

In order to detect OCD symptom severity across time, we performed two distinct linear mixed regression models for the Y-BOCS and OCI-r, respectively. The first linear mixed regression pertained to the changes of OCD-related symptoms detected by the Y-BOCS. We expected to find a significant reduction in the Y-BOCS score across the measurement times. In particular, we expected to find a remarkable difference between the pre-ImRs baseline and the 7-day follow-up. For this reason, in addition to the linear fixed effect of the time, we also estimated its quadratic effect. This allowed us to test whether the differences that had emerged were in the extremes of the experimental region or inside it. Moreover, we expected to find such significant relationships regardless of the randomized duration (i.e., 4, 8, 12, or 16 days) of symptoms monitoring before receiving ImRs. Thus, we estimated the fixed effect considering the time indicator variable as a predictor and the scores obtained on the Y-BOCS at the different detection time, stacked into a single variable, as the criterion. The duration of symptoms monitoring at the pre-ImRs baseline represented the covariate in the model.

**Figure 3:**
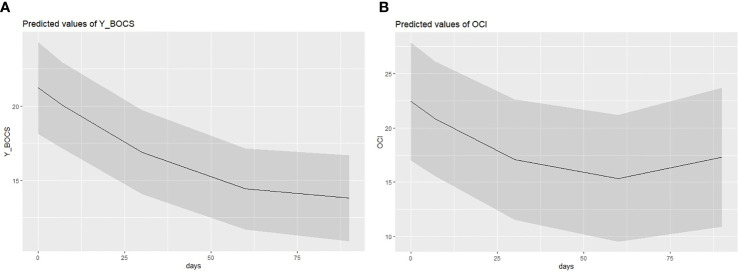
Quadratic fixed effect of time **(A)** on Y-BOCS and **(B)** on OCI-r scores.

Analysis revealed a negative and significant main effect of the time on the Y-BOCS (*B* = −.18; *SE* = .04; *t* = −3.82; *p* <.001; *95%CI* = −.2684, −.0836), which indicated a reduction of the OCD symptoms severity across the different detections (see **Figure 3A**). Moreover, analysis also highlighted a significant quadratic effect of the time (*B* = .001; *SE* = .0004; *t* = 2.11; *p* = .041; *95%CI* = .0004, .0020), suggesting that the stronger differences were to be found in the protocol phases. The more pronounced difference was indeed between the pre-ImRs baseline and the 7-day follow-up. This result was also corroborated by the pairwise comparisons conducted on the estimated marginal means scores of each detection time (see **Table 5A**). The randomized duration of symptom monitoring did not exert any effect (*B* = .01; *SE* =.28; *t* = 0.04; *p* = .97; *95%CI* = −.5893, .6099).

**Table 5A:** Pairwise comparisons based on Y-BOCS estimated marginal means at the different protocol phases.

Days (I) Mean	Days (J) Mean	Mean Diff.	se	p	95%CI	
					Lower	Upper
Pre-ImRs (22.84)	7 days (18.06)	4.78	1.17	.002	1.33	8.23
7 days (18.06)	30 days (17.41)	0.65	1.16	.999	−2.76	4.06
30 days (17.41)	60 days (14.57)	2.84	1.20	.196	−.66	6.34
60 days (14.57)	90 days (14.57)	0.87	1.20	.998	−2.63	4.37

**Figure 4:**
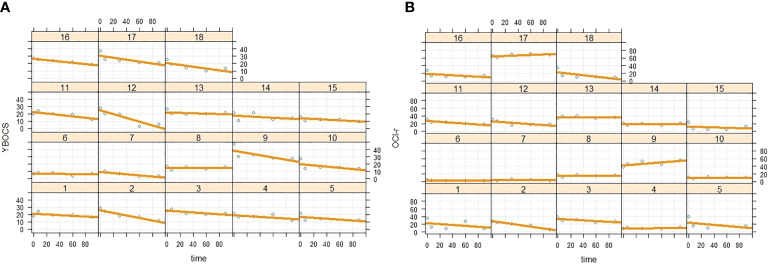
Intercept and slope random variability of time **(A)** on Y-BOCS and **(B)** on OCI-r scores across the 18 patients.

Regarding the random variability of intercept and slope, we found further support for our hypothesis. Analysis yielded a significant random variation of the intercept (*B* = 39.1; *SE* = 15.9; *Z* = 2.45; *p* = .014; *95%CI* = 17.60, 86.98), which simply indicated that patients reported different degrees of OCD symptom severity at the pre-ImRs assessment. More importantly, we also found a nonsignificant random effect for the time slope (*B* = .001; *SE* = .013; *Z* = 0.80; *p* = .423; *95%CI* = −.0001,.0117). Such a result strengthens our result, highlighting how the ImRs intervention produced similar effect across the 18 patients (see **Figure 4A**).

The second linear mixed regression model followed the same procedure as the first, but considering the scores on the OCI-r as dependent variable. Also, in this case, we found results consistent with our expectations. Specifically, analysis showed a negative main effect of the time on the decreases of the OCI-r across the protocol phases (*B* = −.23; *SE* =.08; *t* = −3.15; *p* = .003; 9*5%CI* = −.3910, −.0875), as well as a significant time quadratic effect (*B* = .002; *SE* =.0008; *t* = 2.49; *p* = .016; *95%CI* = .0003,.0037). Graphical representation of the quadratic effect is shown in **Figure 3B**. Note that these coefficients represent unique associations, once the duration of symptom monitoring was checked (*B* = .17; *SE* =.48; *t* = 0.34; *p* = .735; *95%CI* = −.8614, 1.193). The more remarkable reduction of the OCD symptom severity emerged between the pre-ImRs baseline and the 7-day follow-up. Furthermore, in this case the pairwise comparisons supported such result (see **Table 5B**). Moreover, random effects estimates revealed a nonsignificant random variation in the slope (*B* = .002; *SE* = .003; *Z* = 0.69; *p* = .492; *95%CI* = −.0001,.0363), as well as an expected significant variation in the pre-ImRs baseline scores (*B* = 117.18; *SE* = 46.65; *Z* = 2.51; *p* = .012; *95%CI* = 53.70, 255.68) of the OCI-r across patients (see **Figure 4B**).

**Table 5B:** Pairwise comparisons based on Oci-r estimated marginal means at the different protocol phases.

Days (I) Mean	Days (J) Mean	Mean Diff.	se	p	95%CI	
					Lower	Upper
Pre-ImRs (25.35)	7 days (17.46)	7.89	1.93	.002	2.23	13.5
7 days (17.46)	30 days (17.17)	0.29	1.91	.999	−5.29	5.88
30 days (17.17)	60 days (16.40)	0.77	1.97	.999	−4.96	6.50
60 days (16.40)	90 days (16.88)	−0.47	1.97	.999	−6.20	5.25

#### Differences Between High and Low Change at FOGS

In order to clarify the role played by guilt in the change of OCD symptom severity, we implemented two distinct mixed ANOVA on the OCI-r and Y-BOCS. In both analyses, the within factor was thus represented by the scores on these measures at the pretreatment and at the 90-day follow-up measurement times. With regard to the between factor, we split the sample into two subgroups based on the FOGS change score from the prescreening to the 90-day follow-up. Specifically, we computed the differences between the FOGS scores at such phases and then we divided participants based on the sample median value of 5.5. In this way, we obtained low and high FOGS change groups, respectively composed by 8 and 9 participants.

**Figure 5:**
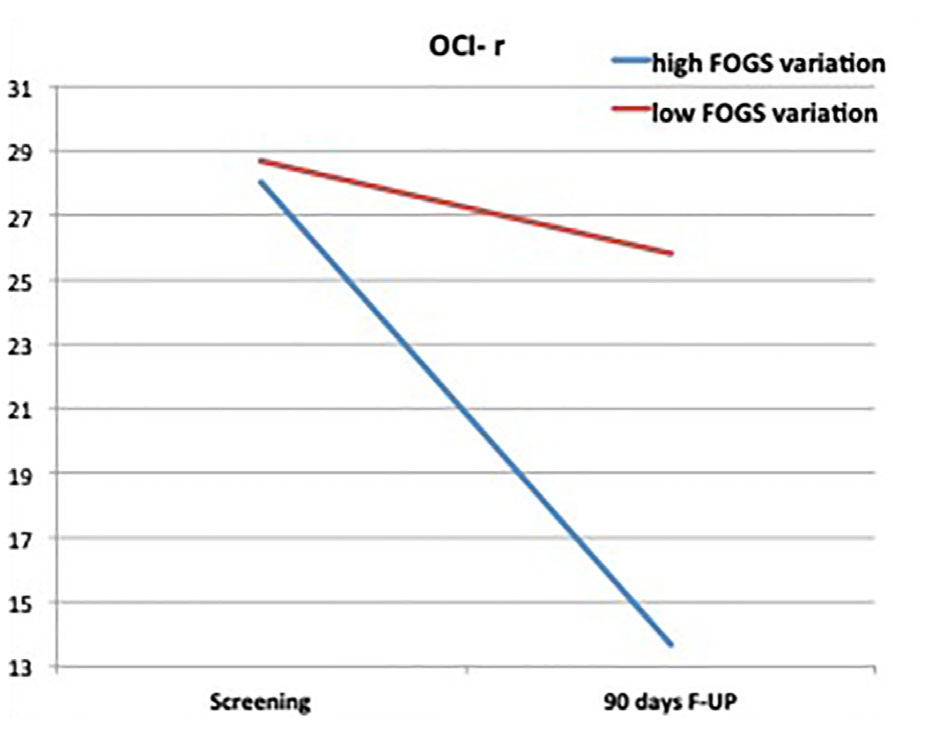
Differences between high and low change at FOGS on the OCI-r.

The mixed ANOVA on the OCI-r showed a significant effect of the treatment (*F[1, 15]* = 8.29, *p* = .01), as well as a significant interactive effect among the within factor and the FOGS change groups (*F[1, 15]* = 7.99, *p* = .01). As can be observed in **Figure 5**, we found a significant decrease in the OCD symptom severity for participants who reported a high FOGS change score (*Mean_Diff_* = 14.22, *se* = 3.42, *p* = .001, *95%CI* = 6.93, 21.51), whereas nonsignificant differences emerged in the group of low FOGS change score (*Mean_Diff_* = .125, *se* = 3.63, *p* = .97, *95%CI* = −7.61, 7.86). Pairwise comparisons also revealed a marginally significant difference between the average score of the two groups at the 90-day follow-up (*Mean_Diff_* = 15.22, *se* = 7.89, *p* = .07, *95%CI* = −1.74, 31.91) and no difference at the pretreatment. These differences were respectively accompanied by standardized effect size (i.e., *Cohen’s d*) equal to 1.7, 0.01, 0.93, 0.07. Consistently, between-subject analyses highlighted a nonsignificant main effect of the FOGS change score factor (*F[1, 15]* = 1.28, *p* = .27).

In contrast, the mixed ANOVA on the Y-BOCS revealed that the decrease on such measure was not moderated by the FOGS change score (*F[1, 15]* = 1.34, *p* = .26), and also that the between-subject effect of this factor was not significant (*F[1, 15]* = 0.77, *p* = .40). In this case, we only found a significant within-subjects main effect of the treatment, which showed that the Y-BOCS scores decreased similarly for participants with both high and low change on the FOGS (*F[1, 15]* = 37.99, *p* <.001).

#### Intercorrelations Among Measures at the 90-Day Follow-Up

Finally, in order to observe the correlations among the measures involved in the study, we computed correlations between all the outcome variables at 90-day follow-up. As can be seen in **Table 6**, we found significant correlation among most of the interested variables. Specifically, we observed a positive association between OCD symptomatology (both assessed with Y-BOCS and OCI-r), BDI, BAI and DPQ, whereas we witnessed that the FOGS was only positively related with the OCD symptomatology assessed by OCI-r.

**Table 6:** Intercorrelations among the measures assessed at the 90-day follow-up.

	1	2	3	4	5
1. Y-BOCS					
2. OCI-r	.76**				
3. FOGS	.30	.67**			
4. DPQ	.51*	.70**	.33		
5. BDI	.61**	.53*	.21	.66**	
6. BAI	.62**	.54*	.17	.43	.81**

## Discussion

The main result of our study is that after three ImRs sessions on guilt-inducing memories, OCD participants experienced a significant clinical reduction of symptoms.

Comparison between OCD measures at screening and at the 90-day follow-up indicates a significant clinical improvement in symptomatology, in terms of greater management of thoughts and obsessions, less time occupied and less interference in general, greater control over compulsions and an increased awareness of discomfort and exaggerated thoughts, as assessed by Y-BOCS. At the 90-day follow-up, the total OCI-R scores significantly diminish, the average scores from pretreatment and post-treatment did not meet the clinically reliable change criteria.

The difference between Y-BOCS and OCI-R is not surprising, in fact it is in line with the work of Abramowitz and Deacon (6), which found a low correlation between the OCI-r and Y-BOCS severity scores in a group of OCD patients. Sulkowski et al. (90) suggested that this might be because of the differences in symptom “coverage” by the OCI-r and Y-BOCS as stated by Maloney and colleagues who concluded that “*the clinician-administered Y-BOCS and the self-report OCI capture different aspects of symptomatology or of its improvement*” [(57), p. 6].

Our results are in line with the study by Veale et al. (54) and Maloney et al. (57). ImRs confirms its promising application in treating OCD, underlining the importance of working on memory of past events. Veale et al. (54) and Maloney et al. (57), who focused on past aversive memories emotionally linked to present recurrent, intrusive and distressing images, detected through Speckens’s interview (49). We, instead, chose to focus on childhood memories of guilt-inducing reproaches, detected while asking the participants to remember events characterized by features found by Basile et al. (46). What is interesting is the meaning attached to the memories selected by Veale et al. (54), which, in many cases, related to a negative moral judgement about the self. The beliefs reported by participants in the work of Veale et al. (54), are similar to those reported by the participants of this study, even though the memories are somewhat different. This may suggest that the interpretation of guilt-inducing reproaches may be similar to how future OCD patients interpret even other experiences. Although the memories selected in this study were not necessarily related to current symptoms, they may have been related if they were associated with intrusive images.

It is interesting to observe that all the participants found events characterized by the features found by Basile et al. (46). All the participants remembered having felt, during the episodes, an intense sense of guilt and having thought themselves to be a “bad person.” Looking at **Appendix Table 1**, subjects reattribute the causes of what happened to something external and not to their own wickedness, worthlessness, lack of ability, or defect. In particular, for the case of the reattribution of culpability, the new meaning after intervention is more flexible and participants recognize that the fault committed was not so serious or that they too have the right to make mistakes. As proposed by Arntz (53) ImRs confirms its efficacy in changing the meanings attached to past adverse events in childhood or adolescence.

ImRs, as hypothesized, showed a significant reduction of the fear of guilt. Interestingly, participants who showed a higher reduction in fear of guilt displayed a higher reduction in obsessive symptoms, when assessed by OCI-R. This result suggests that fear of guilt moderates the ImRs effect on obsessive symptoms and this effect is consistent with the hypothesis that fear of guilt plays a central role in the onset and maintenance of OCD symptoms (18, 91).

ImRs reduced the disgust propensity, but in a marginal way, less intense than what was hypothesized, as ImRs doesn’t directly targeted disgust.

The intervention on the memories of guilt-induced reproaches reduced depression and anxiety in a statistically significant way. A similar result was observed in the work of Maloney et al. (57). However, the improvement did not meet the criteria for reliable and clinically significant change. At the last follow-up a correlation was observed between anxiety and depression and OCD symptoms when assessed by Y-BOCS. The reduction in anxiety is easily understandable, since very often this emotion accompanies obsessive symptoms. Zandberg et al. (92) found a reduction in depression following the improvement in obsessive symptoms. This is understandable considering that, in many cases, depression is related to the frustration and distress of having obsessive symptoms. For example, obsessive symptoms may involve a reduction in interpersonal relationships and may also produce a reduction in self-esteem and self-effectiveness.

The present work sheds light on the role that repeated experiences of criticism, and consequent guilt induction, might play in the genesis of dysfunctional beliefs about the self that are related to OCD development. This evidence should encourage clinicians to consider the role of sensitizing experiences in OCD treatment, addressing guilt-specific intervention.

### Limitations

The findings of this study must be viewed in light of some limitations.

The main limitation of this study is the small sample size. A larger sample would allow an evaluation specifically separated by subtypes, to test whether ImRs on guilt-inducing memories of reproaches shows the same result in all OCD subtypes. Certainly, considering that our study, together with the study by Veale et al. (54) and the study by Maloney et al. (57) that assess the efficacy of ImRs in OCD, it may be worth investing more resources to conduct a randomized controlled trial study. Another limit of this study is related to the absence of a control group, in fact, without it, we cannot exclude that ImRs on guilt-inducing reproaches is not effective in other disorders, for example in social phobia, and therefore its effect in OCD is nonspecific. In addition, we are unable to say whether ImRs on memories noncharacterized by guilt-inducing reproaches, such as abandonment, can be equally effective in treating obsessive symptoms.

Moreover, the study is missing multiple assessments carried out in different phases and between sessions. The intensity of beliefs and emotions that were connected to the episode targeted in ImRs were not measured.

### Future Directions

Assessing the effectiveness of ImRs on memories of guilt-inducing reproaches for participants with different disorders may be carried out, with the aim of understanding the similarities and differences between the effect of sensitizing experiences with OCD participants. When considering OCD, future research could consider randomized controlled trials, comparing the effect on OCD symptoms of ImRs on guilt-inducing memories, of other techniques aimed at changing the emotional valence of memories and comparing the effect by selecting memories with other emotional valences.

This research supports the importance of taking into account work on the historical vulnerability of OCD in CBT. In line with this proposal, recent work (93) has offered the first suggestion of an integration between CBT and Schema Therapy, aimed at reducing OCD’s historical vulnerability. However, further studies on techniques aimed at changing this vulnerability are necessary.

## Data Availability Statement

The raw data supporting the conclusions of this article will be made available by the authors, without undue reservation.

## Ethics Statement

The studies involving human participants were reviewed and approved by Ethical committee of Guglielmo Marconi University. The patients/participants provided their written informed consent to participate in this study. Written informed consent was obtained from the individual(s) for the publication of any potentially identifiable images or data included in this article.

## Author Contributions

KT, BB, TC, BS, SF, AG, OIL, CP, GR, and AMS carried out the interventions. KT wrote the manuscript with support from BB and VP, who analyzed the data. GF contributed to sample preparation and FM conceived the original idea and supervised the project. All authors contributed to the article and approved the submitted version.

## Conflict of Interest

The authors declare that the research was conducted in the absence of any commercial or financial relationships that could be construed as a potential conflict of interest.

## APPENDIX

**Table A1:** Summary of the contents of the rescripted memories.

Participant	Rescripted memory	Emotion and meaning of memory	Age in ImRs	New meaning after ImRs	
1	Mother accuses the patient of being stupid and gets very violent toward him.	Guilt, fear and sadness. *“I think I’m a bad child”*	*6*	*“Mom had serious mental health problems.”*	
2	At home, scolded and beaten by his parents for responding negatively to his mother.	Guilt and anger “I am bad”/”They are unfair”	7	*“It was not my fault, I was just a kid, they were disturbed.”*	
3	He throws tantrums and doesn’t want to study. Parents threaten to abandon him in a Gypsy camp.	Guilt and fear. *“It’s my fault because I do not do well at school, I’m incapable”*	13	*“I am not incapable if I do not do well at school and the punishment is exaggerated.”*	
4	The father scolds him violently for dropping a candle, causing a fire to start in the hall without realizing it (and mother doesn’t intervene).	Guilt. *“I am irresponsible and dangerous and I don’t deserve affection. I must learn to always be careful”*	9	*“I’m a valuable and lovable person.”*	
5	At school playing with a friend, he throws a toy hitting his friend’s head and watches his reaction. When he realizes that his friend is crying and has a cut, he worries and gets scared and goes into the corner.	Guilt. *“I did something serious and irresponsible. I could have hurt her so much. Maybe I even enjoyed doing it, because as she cried I looked at her with satisfaction. I’m a gross person.”*	5	*“It was just an accident. It can happen when you are 5 and I immediately realized I made a mistake, it’s understandable when you are 5.”*	
6	The father scolds him after finding him playing naked with a friend.	Guilt. “*I’m wrong and dirty.”*	9	*“I’m not dirty. Dad is wrong.”*	
7	The parents don’t allow her to have a cat, and she thinks she has abandoned her cat.	Guilt “*The cat could die because I didn’t do enough to convince my parents to bring him with us”; “My mom screams at me because it’s not appropriate to bring a cat”. “I’m a bad girl. I am unlovable”*	5	*“I am heard (my opinion counts); I am good the way I am; I am loved.”*	
8	The mother scolds her for listening to music while studying.	Guilt and anxiety*; “I was wrong and cannot make up for it! I have disappointed my mother!”*	12	*“I have the right to be loved unconditionally. I’m ok the way I am. I don’t necessarily have to satisfy high-level expectations.”*	
9	After having talked to the teachers, the father scolds her for having been too bossy with her classmates.	Guilt, sadness *“I have done everything possible, and I am still not ok”*	9	*“I am ok, no matter what I do.”*	
10	His father is embarrassed after he comments on a soccer player’s appearance.	Guilt, anxiety *“I cannot think whatever I want”*	9	“*I am not wrong.”*	
11	Her mom is disappointed because she did not get the best mark at school.	Guilt *“I feel like a failure and I think I made a big mistake”*	*8*	*“Mom’s standards are too high and it’s not me being too needy, I am just a child.”*	
12	She is with her dad, and she is learning to write. She makes a mistake, and her dad gets very angry and threatens to beat her.	Guilt *“I am scared of making mistakes and of my dad’s violent reaction”*	*6*	*“Dad has serious mental problems, and I am just a kid. I have the right to make mistakes, and this does not mean I deserve to be punished or beaten.”*	
13	The mother scolds and slaps him for having bought a book with his savings: “Y*ou should be ashamed, while your father sweats blood to provide for this family, you waste money on this foolishness!”*	Guilt, shame and fear over financial issues. “*I’m bad and selfish, I damaged my family financially to satisfy my whim”*	9	*“She should understand my needs as a child and not dump her anxiety and the problems of the entire family on me.”*	
14	The mother scolds her because she secretly throws out the meat that she was forced to eat. She is forced to confess in front of her sisters that she has disobeyed and lied.	Guilt, shame, anger. *“I’m a bad person”*	8	*“I have the right to be listened to about my preferences, needs, and demands. I’m not a bad child and I can express my needs.”*	
15	The mother scolds her for breaking a lamp she loved. She says: “*Curse you! I curse your life as you have ruined mine!”*. This was followed by a long face that lasted for days.	Guilt and fear of ruining the relationship with her mother. *“I’m bad; mom will not want anything to do with me”*	7	*“My mother was always angry, she had some serious mental problems, she shouldn’t have said those things to her children because children believe them. I was a normal child and she made me believe that I was a wicked monster.”*	
16	Mother scolds him for lying.	Guilt and fear of punishment; *“I’m a bad person”*	9	*“I am not bad: all children lie sometimes.”*	
17	At home. Scolded and beaten by her mother for doing her homework too slowly.	Guilt, shock, fear, *“What happened? Why does she do this?/My God, she will end up hurting me! I must have done something wrong”*	8	*“I did not do anything wrong. I am ok. Her behavior was wrong and insane.”*	
18	He feels responsible for defending his brother from the aggressiveness of his father.	Guilt, impotence and fear *“I do not know what I should do, and I am afraid of not being able to defend my brother”*	14	*“It is not my responsibility, I have to think about me, and my brother knows how to defend himself from Dad.”*	

## APPENDIX

### Procedure to Detect Guilt-Inducing Reproach Memories

The target of the intervention is memory of:

- Guilt
- Hypo-/Hyper-responsibility
- Observation of the guilt-inducing experiences on someone else
- Have caused real harm
- Responsibility for choosing the right partner

#### Interview on Guilt-Inducing Memories

**PLAN A (Direct questions to the patient, examples are requested):**

- Did you often feel guilty as a child?
- As a child, were you afraid you might be guilty of something?
- Have you ever caused anyone real harm?
- As a child, did you feel responsible for the well-being of someone close to you?
- Were there times when you felt you were not up to your responsibilities?

Questions about the family of origin:

How old is your father? What is/was your father like? Give me three adjectives to describe him.
When you were a child, did your father make you feel guilty? (ask for examples)
Were you afraid of being scolded by your father? (ask for examples)
When you were a child, did your father often scold you or someone close to you? (ask for examples)
The same procedure with mother or other significant figures (e.g., grandparents)

NOTE: WHEN THE PATIENT PROVIDES MORE THAN A MEMORY OF EQUAL RELEVANCE, CHOOSE THE OLDEST, THAN THE MOST PAINFUL.

**PLAN B (Imagery assessment):**

We ask the patient, using his/her imagination, to describe the last time she/he felt guilty.
Through the floating back, using emotion and mental status as a bridge, we ask her/him when she/he felt this way as a child.

NOTE: TO AVOID PROLONGED EXPOSURE OF THE PATIENT TO THE MEMORY, ONCE THE MEMORY HAS BEEN RECALLED, ASK THE PATIENT TO DESCRIBE THE MEMORY FACE TO FACE.

**PLAN C (Scripts of memories):**

We ask the patient to read some prototype memories and ask whether she/he has similar memories:

Guilt-inducing
  When Peter was in elementary school, his father usually accompanied him to school. Despite his efforts, Peter had some difficulty getting out of the house on time and his father often became furious. Peter remembers the time when his father went on a rant in a loud and angry voice, with an outraged expression on his face and without looking him in the eye, while tinkering with the car keys to start the car: “The daily drop of poison!” and, “Life is good, huh. Who cares if your dad wakes up at 6:00 every morning to drive you to school on time then runs to work to support the family and allows you and your sister to study! This is the thanks I get!” After that the father did not speak for the rest of the day. At that moment, Peter was stunned, intimidated and mortified. What most distressed him was that he faced at least five hours of school, knowing he would feel a kind of boulder in his stomach and could not do anything to remedy it.
Hyper-responsibility
  When Albert was nine years old, his mother had a health problem and his father was often out of the house because of work. He was considered by all to be a very responsible child for his age.
  He was very diligent, taking his two younger sisters to school when their mother couldn’t get out of bed. He also prepared lunch for his sisters and reminded his mother to take the medication the doctor prescribed. Albert put others’ need ahead of his own. For example, he once gave up going to his best friend’s birthday party, which he had been looking forward to for a long time, because his mother was in bed with a headache and he wouldn’t leave her alone.
Observation of someone else’s guilt-inducing experiences
  Anna has a sister named Silvia, who is seven years old and attends elementary school.
  Once, Silvia got a teacher’s note saying her behavior was too lively in class. When she came home, Silvia didn’t tell her parents, and wanted to keep the note hidden from her father.
  At some point, she confessed it to her mom, who told her dad. Anna saw the father who, having “discovered the lie,” got very angry and screamed at Silvia in an aggressive and contemptuous tone: “You have a guilty conscience, like a panty dirty with poo. You are not sincere, you are not truthful.” Anna saw Silvia burst into tears and felt a strong sense of guilt, for both the note and the lie, and for not knowing how to remedy the situation.
Having caused real harm
  Maria is eight years old and has a younger sister and often plays catch in her arms, pretending to be her mother. Maria loves her sister very much and her mother is very happy with the relationship that is being established between the two sisters. One day, while she is in the kitchen with her mother, Maria takes her little sister in her arms and tenderly begins to play with her. The little one, unpredictably, waddles, falls to the ground and starts to bleed from the lip. The mother rushes to rescue the little one and says nothing to Maria, who is frightened by the blood that she sees dripping from the baby’s lip and feels deeply guilty for harming her sister.
Relationship OCD
  Mario remembers how worried he was when his parents got divorced. Every night, for example, and during the night, he would go to the bedroom to check that his parents were sleeping together. They argued daily, and the arguments were often very heated and ended most often with the mother crying and the father slamming the door. In these discussions Mario took sides with his mother, was angry with his father, and thought that he had married her only for economic convenience, not because he loved her, since he humiliated her constantly. The mother suffered greatly from the father’s attitude toward other women and toward the family; attentive and seductive toward the former, cold and detached at home. Mario recounts this episode: the whole family was at the table with his mother’s cousin, her husband and their two children. He remembers how his father was full of attention toward his mother’s cousin: “He changed physically, almost became taller, the tone of voice, his eyes, his face, his smile, all bent to seduce, not caring that my mother and all of us were there.
  It was really humiliating for her and I felt anger and disgust growing inside me.”
Hypo-responsibility
  Antony is 18 years old and has recently left his small village of origin to move to a large city to attend university. It was a big change: Now Antony has to take care of the house, do the shopping, and also devote himself to his studies. One day, when there are only a few days left before the first exam, Antony realizes that he has completed only half of the planned program. At that moment Antony feels unable to face his responsibilities and remembers how his mother protected him all the time and how, when he was at home with her, he was relieved not to have any responsibilities.
